# 5G Network Coverage Planning and Analysis of the Deployment Challenges

**DOI:** 10.3390/s21196608

**Published:** 2021-10-03

**Authors:** Md Maruf Ahamed, Saleh Faruque

**Affiliations:** 1 Department of Electrical and Computer Engineering, Iowa State University, Ames, IA 50011, USA; 2 School of Electrical Engineering & Computer Science, University of North Dakota, Grand Forks, ND 58202, USA; saleh.faruque@und.edu

**Keywords:** 5G, 5G coverage, antenna array, massive MIMO, millimeter-wave, SINR

## Abstract

The 5G cellular network is no longer hype. Mobile network operators (MNO) around the world (e.g., Verizon and AT&T in the USA) started deploying 5G networks in mid-frequency bands (i.e., 3–6 GHz) with existing 4G cellular networks. The mid-frequency band can significantly boost the existing network performance additional spectrum (i.e., 50 MHz–100 MHz). However, the high-frequency bands (i.e., 24 GHz–100 GHz) can offer a wider spectrum (i.e., 400~800 MHz), which is needed to meet the ever-growing capacity demands, highest bitrates (~20 Gb/s), and lowest latencies. As we move to the higher frequency bands, the free space propagation loss increases significantly, which will limit the individual cell site radius to 100 m for the high-frequency band compared to several kilometers in 4G. Therefore, the MNOs will need to deploy hundreds of new small cells (e.g., 100 m cell radius) compared to one large cell site (e.g., Macrocell with several km in radius) to ensure 100% network coverage for the same area. It will be a big challenge for the MNOs to accurately plan and acquire these massive numbers of new cell site locations to provide uniform 5G coverage. This paper first describes the 5G coverage planning with a traditional three-sector cell. It then proposes an updated cell architecture with six sectors and an advanced antenna system that provides better 5G coverage. Finally, it describes the potential challenges of 5G network deployment with future research directions.

## 1. Introduction

With the advancement of every generation (i.e., starting from the voice-only systems to today’s intelligent communication systems), the mobile network introduces new use cases and services, shown in Table 1 [1,2,3,4]. Until the 5G networks, all the new use cases and services were introduced to attract more human users to the mobile network. However, the 5G networks open up a new horizon and promise that the mobile network will be used for human-centric applications. It will also interconnect billions of smart devices autonomously while ensuring security and privacy [5,6]. The 5G network will enable the emerging services that include remote monitoring and real-time control of a diverse range of smart devices. It will support machine-to-machine (M2M) services and Internet of Things (IoT), such as connected cars, connected homes, moving robots, and sensors, etc. [7,8].

**Figure 1:**
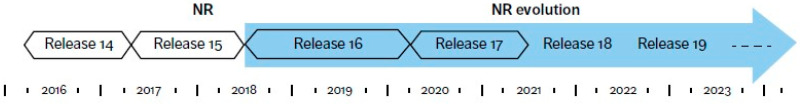
5G NR evolution time plan.

The 5G network evolution is well underway, and it has progressed swiftly since the 3GPP standardized the first 5G NR (New Radio) release (release 15) in mid-2018, shown in Figure 1 [9]. The leading mobile network operators (MNOs) in several regions of the world have already launched the first commercial 5G NR networks with mid-bands (i.e., 3–6 GHz) with the existing 4G cell sites, resulting in a significant performance boost [9,10,11].

However, the 5G network is projected to reach 40 percent population coverage and 1.9 billion subscriptions by 2024, corresponding to 20 percent of all mobile subscriptions [8]. These figures indicate that it will be the fastest global rollout so far. Moreover, smartphones generated data traffic is about 90 percent and is estimated to reach 95 percent by the end of 2024 [10]. With the continued growth of smartphone usage, the worldwide mobile data traffic is predicted to reach about 130 exabytes per month, which is four times higher than the corresponding figure for 2019, and 35 percent of the traffic will be carried by 5G networks [11,12]. This ever-growing data demand and massive data traffic requirements can be met with the additional spectrum offered by the high-bands (i.e., 24 GHz–40 GHz) of 5G NR (new radio). The current 4G network can offer the carrier bandwidth from 5 MHz, 10 MHz, and 20 MHz, but the 5G mid-bands can offer 50 MHz and 100 MHz, whereas the 5G high-band can offer 400~800 MHz [12]. Since the wider band provides a shorter transmission time interval, it will enable lower network latency [13].

**Figure 2:**
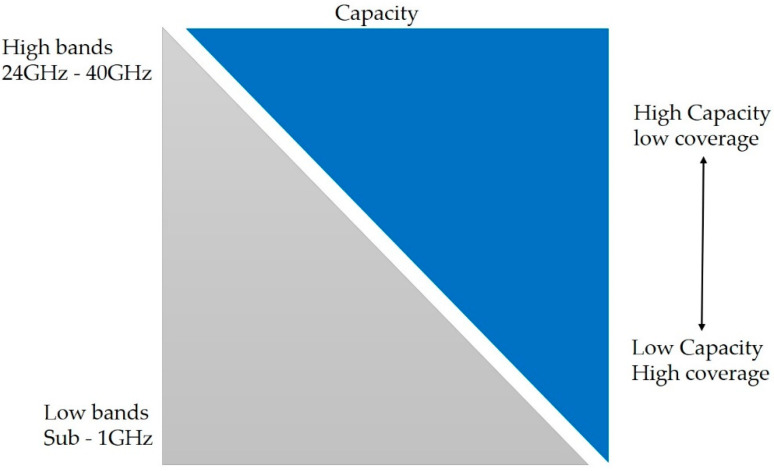
Coverage and capacity characteristics of 5G radio frequency ranges.

The 5G NR deployment in high bands (i.e., 24 GHz–40 GHz) offers more spectrum, which can boost the network capacity and the data rate by several folds [14]. In higher frequency bands, the signal propagation loss is too high; that will significantly reduce the individual cell site coverage, as shown in Figure 2 [13,15]. The individual cell site coverage will be around 100 m (in radius) in higher frequency bands (i.e., 24 GHz–40 GHz) compared to several kilometers in 4G networks [14,16]. Therefore, the MNO must deploy hundreds of new small cells (e.g., 100 m cell radius) compared to one large cell site (e.g., Macrocell will have several km in radius) to ensure the end-users’ connectivity for the same area. The 5G network will require a very high level of network availability (i.e., 99.999%) and very high network reliability (i.e., 99.99%) that will require 100% network coverage [4,17,18], meaning that the 5G network coverage planning will be one of the main priorities for the MNO. It will ensure 5G connectivity and meet all the 5G network key performance indicators (KPI) (e.g., 99.99% network availability, 99.99% network reliability, highest bitrates (~20 Gb/s), and lowest latencies, etc.).

To deploy the 5G network and test the KPI of the networks for any deployment areas, the MNO will have the following two options:

1. The first option begins with deploying all the required cell sites without prior analysis (e.g., each cell site location and cell site parameters, etc.). Then test the radio signals disperse throughout the deployment area and update the cell site parameters (e.g., antenna height, transmit power, power supply, backhaul connections, etc.) accordingly to meet the 5G network KPI requirements. If there is any coverage gap/hole, they deploy additional small cells to meet the coverage requirements. However, every time a new small cell is deployed in any area, the MNO must test the radio signal again to verify the 5G KPI requirements and identify the potential cell site interferences.
2. The second option begins with developing a 5G network simulation environment with an appropriate terrain profile and the cell site parameters. Then, analysis of the radio signals is conducted throughout the deployment area. The radio signal study will help the MNOs to identify the accurate cell site location and the appropriate cell site parameters for each cell. Finally, all the required cell sites are deployed for any particular area and the radio signal throughout the deployment area is tested to verify the 5G network KPI.

The MNO will need to deploy hundreds of new small cells instead of one large Macrocell to ensure the full network coverage for any particular area. It will be a major challenge for the MNO to identify and place each new cell site in an accurate location with appropriate cell site parameters (e.g., antenna height, transmit power, power supply, backhaul connections, etc.) without prior analysis. Therefore, the second approach can save a lot of time and cost for the MNO.

The contributions of this article are summarized below:

- This paper’s first scope is describing the 5G network coverage planning. It includes the 5G RAN (Radio Access Network) network layout planning, selecting cell site parameters (e.g., design transmitter sites, cell site height, cell site location, operating frequency, transmit power, etc.), and selecting the propagation model for the accurate measurement.
- This paper’s second scope is to analyze the RF coverage (e.g., SINR and signal strength) of the traditional three-sector cell architecture. It then proposes an updated cell architecture with a six-sector cell that provides better RF coverage with appropriate antenna parameter settings (e.g., antenna downtilt angle and antenna height).
- This paper’s third scope describes the 5G network deployment challenges and provides guidelines for overcoming these challenges.

The rest of the paper is organized as follows: Section 2 describes the 5G network planning (e.g., define network layout, design transmitter sites, select propagation model, etc.) and test environment. Section 3 presents the 5G network coverage and SINR simulation results. Section 4 describes the 5G network deployment challenges with future research directions, and the conclusions are discussed in Section 5. 

## 2. 5G Network Coverage Planning

The 5G network will require 100% network coverage to meet the high network availability (i.e., 99.999%) and network reliability (i.e., 99.99%). Therefore, the optimum network coverage planning will be the key to ensure all the network requirements of 5G and provide the requested services to the users. This section first describes the fundamental of network coverage. It then describes the overall network coverage planning for 5G, including network layout planning, transmitter site designing, and propagation modeling. 

### 2.1. What Is Network Coverage?

**Figure 3:**
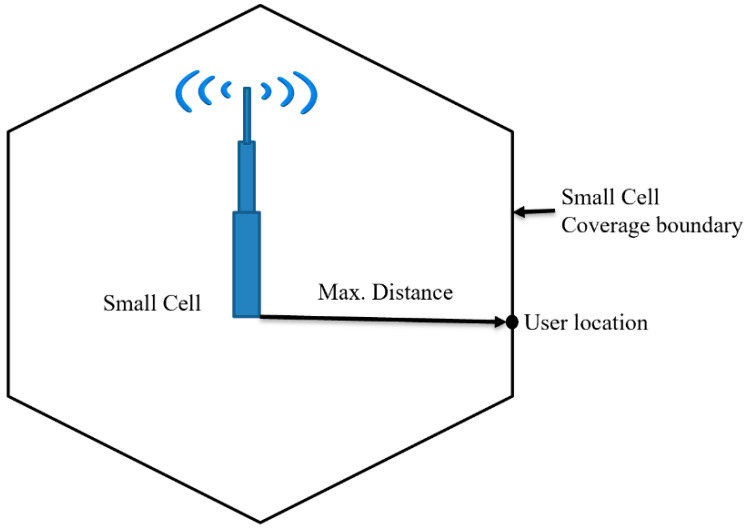
Small Cell network coverage.

The network coverage refers to the area around the base station/cell site where the user can send any service requests and successfully connect with the cell site to receive the services. We calculate the maximum distance from the cell site to the user from where the user can send any service requests and receive the services without interruption, called the radius of the cell, shown in Figure 3. The cell radius defines the maximum cell coverage/cell coverage boundary, and the user will not establish any connections beyond the cell boundary. Once we calculate the cell range, we can estimate the number of cell sites required to cover a deployment area.

### 2.2. Define Network Layout

**Figure 4:**
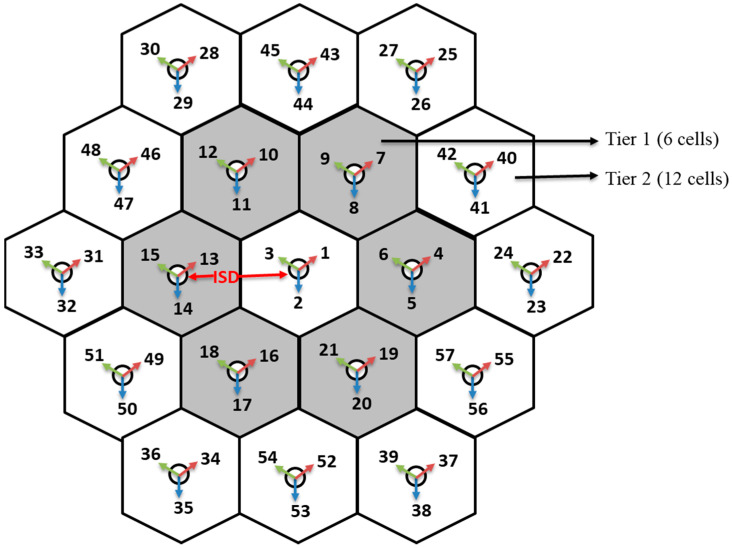
5G Network layout that has 19 cells, each with three sectors.

In higher-frequency bands, the individual 5G small cell site will have 100 m (in radius) cell coverage. When the MNOs need to deploy more than one small cell to cover the deployment area, the adjacent small cell location must maintain a certain distance called inter-site distance (ISD), shown in Figure 4 [16]. The users can get more than one dominant receive signals from the nearby cell sites if the minimum ISD is not maintained, called out-of-cell interference, which can significantly degrade the network performances. This paper considers 100 m cell radius and 200 m ISD to avoid out-of-cell interferences. 

Since the MNOs need to deploy hundreds of cells to cover a deployment area/region, they must find a better way for faster 5G deployment. This paper first creates a group of cells with three sectors, where the first tier has 6 cells, the second tier has 12 cells, the third tier contains 18 cells, and it can be increased even further based on the deployment area size. Once the group of cells has been created, it can be reused based on the deployment area size. This paper considers the dense urban environment for the network planning and coverage analysis. In the test environment, the network layout consists of 19 sites (i.e., tier 2) placed in a hexagonal layout, each with three sectors, as shown in Figure 4. 

### 2.3. Define Transmitter Sites

Three transmitters correspond to each cell sites, as shown in Figure 4. Each transmitter can be designed with a single antenna element or antenna array that contains n numbers of antenna elements. Since the 5G network will use the higher frequency bands (i.e., millimeter-wave bands), it will allow integration of a large number of antennas in a single array (e.g., 8-by-8 rectangular array) with a small form factor. According to IMT-2020 [16], the 5G base station can have up to 256 antenna elements with an operating frequency of 30 GHz. 

#### 2.3.1. Design an Antenna Array

**Figure 5:**
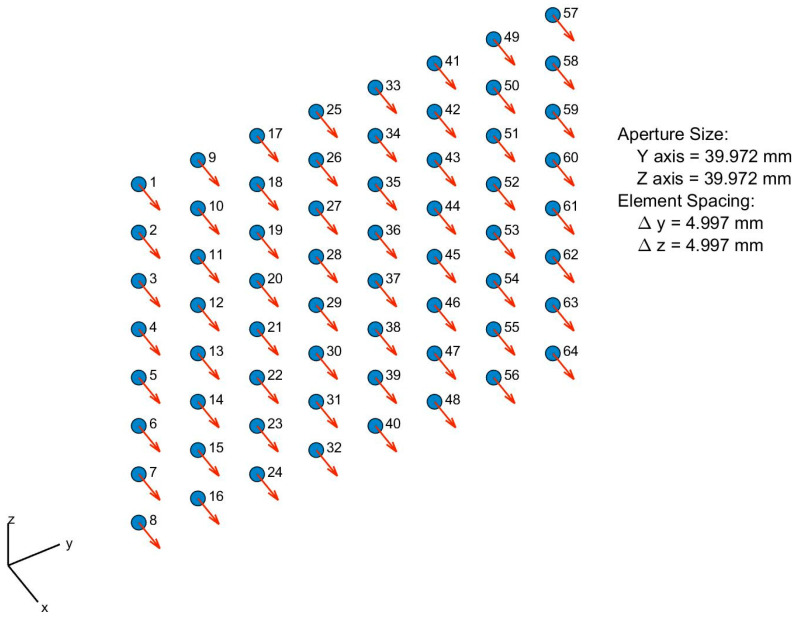
An 8-by-8 uniform rectangular array.

**Figure 6:**
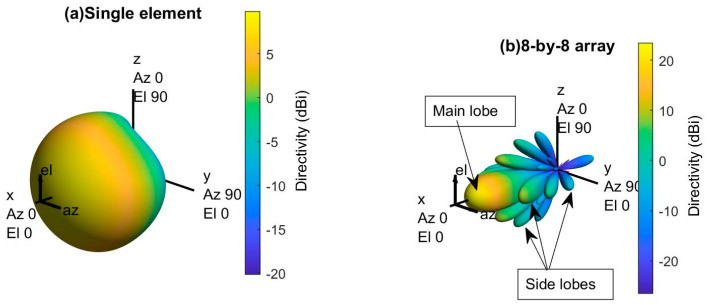
3D radiation pattern of a single element and an 8-by-8 antenna array for 30 GHz operating frequency.

An antenna array (or phased array) is a set of two or more connected antennas which work together as a single antenna to transmit or receive radio waves, shown in Figure 5 [19,20]. Typically, the single element antenna provides a relatively wide radiation pattern that is low in gain (directivity), but the radiation pattern of an antenna array provides a narrower beam, and it can achieve higher gain (directivity), which is required for long-distance communications [19]. This paper uses the base station antenna characteristics defines by the ITU-R report for 30 GHz operating frequency [16]. Figure 6 shows the 3D radiation pattern of a base station with a single element and an array with 8-by-8 antenna elements. Several MATLAB toolboxes (e.g., Antenna Toolbox, Phased Array System Toolbox, etc.) are used for the Antenna design and simulations. 

The antenna array provides path diversity (also called MIMO), and the path diversity increases with the sizes of the array [19]. The 5G networks will use the larger antenna array (~256 antenna elements) on the transmitter (i.e., cell site) and receiver (i.e., mobile phone) to take advantage of the massive MIMO, which will increase the network reliability [21,22]. 

The phased array also allows changing the phase of each element electronically, which can be used to steer the radio beam in a different direction to avoid interference coming from a specific direction [20]. This paper shows that the signal-to-interference- plus-noise ratio (SINR) of 5G networks can also be improved by using the larger size array for the individual cell site transmitters. 

It is unnecessary, but it is often convenient and more practical to use identical elements in the array. The radiation pattern of such an antenna array depends on the following factors [19]:

1. The geometrical configuration (e.g., linear, circular, rectangular, etc.) of the array.
2. The relative spacing between the elements.
3. The relative radiation pattern of each element.
4. The excitation phase and amplitude of the individual elements.

This paper considers a uniform rectangular phased array that uses identical elements and uniform spacing between elements.

#### 2.3.2. Antenna Array with Reduced Grating Lobe’s 

The vector addition of the radio waves radiated by the individual antenna elements determines the array’s radiation pattern. The radiated waves of each individual element interfere constructively in one direction that produces a strong beam in one direction called the main lobe, shown in Figure 6. However, all the radiated waves interfering destructively cancel out; that reduces the powers radiated in other directions. However, the uniform array (i.e., arrays with uniform spacing between elements) will have a series of weaker radiated beams at different angles than the main lobe called sidelobe (shown in Figure 6), and these sidelobes are undesirable in most array applications [19,20,23]. 

**Figure 7:**
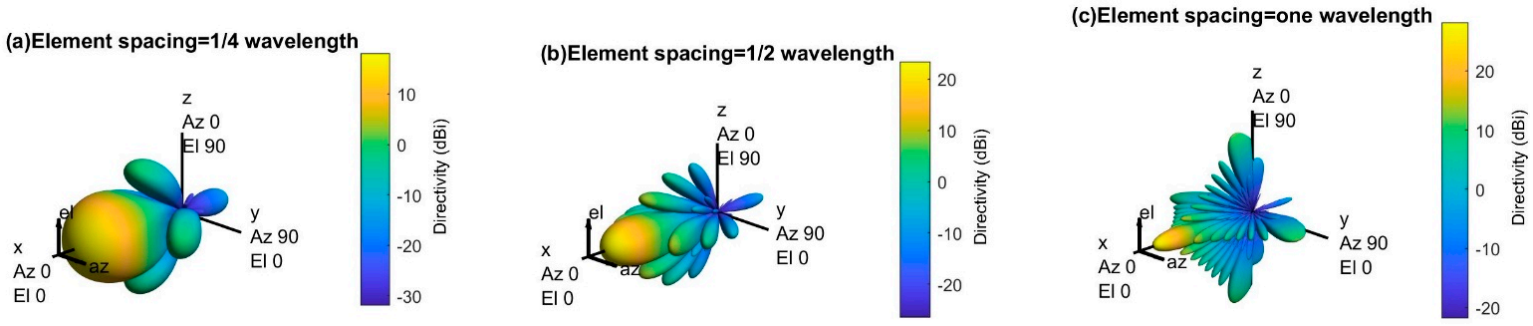
3D radiation pattern of an 8-by-8 antenna array with different element spacing for 30 GHz operating frequency.

Figure 7 shows the 3D radiation pattern of an 8-by-8 uniform phased array with different spacing (i.e., one-quarter wavelength, one-half wavelength, and one wavelength) between elements. The main lobe becomes more directive (high gain) as the element spacing increases, but the number of sidelobes and sidelobe power increase. The number of sidelobes and the sidelobe power must be reduced to direct the maximum power toward the main lobe. 

There is more than one strong beam (i.e., main lobe) in Figure 7c, and these unintended beams in different directions are called grating lobes. Since the 5G network requires directional array antennas that radiate in a specific direction to avoid the out-of-cell interferences, the grating lobes must be avoided for better network performance. However, there are no grating lobes when the element spacing is less than or equal to one-half of wavelength, as shown in Figure 7a,b. Therefore, this paper considers one-half wavelength element spacing for the uniform phased array. 

#### 2.3.3. Antenna Array with Reduced Sidelobe’s Power

**Figure 8:**
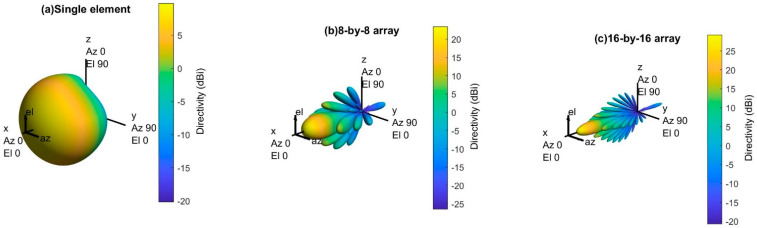
3D radiation pattern of a single site with different array size.

Figure 8 represents the 3D radiation pattern of a single transmitter with different antenna array sizes (e.g., 8-by-8 array and 16-by-16 array). The antenna radiation becomes more directive (i.e., high gain) as the number of elements in the antenna array increases. Since the directional gain increases with the size of the antenna array, it will also increase the peak SINR (signal-to-interference-plus-noise ratio). Eventually, it will improve the overall network performance. 

However, increasing the number of antenna elements per array also increases the number of sidelobes, and the sidelobe power significantly, shown in Figure 8b,c. Although the larger array opens the opportunity for the massive MIMO, it also comes with a new challenge, which is sidelobe power. The sidelobe power must be reduced before implementing this transmitter with any base stations.

There are several amplitude tapering (e.g., Dolph–Chebychev, Taylor, and Binomial) methods to reduce the sidelobe power. The array elements are tapered maximum at the center and minimum at the edges [24,25]. This paper uses the Dolph–Chebychev amplitude tapering method to reduce the sidelobe power. 

Figure 8 shows that the largest sidelobes are closer to the main lobe and the smallest sidelobes are far away from the main lobe. A window function can be designed to achieve a minimum sidelobe power of R dB (e.g., 30 dB), where the sidelobes closer to the main lobe will be attenuated by R dB (e.g., 30 dB). However, the sidelobes far away from the main lobe will be attenuated higher than R dB (e.g., 30 dB). This optimum window is known as the Dolph-Chebyshev window, and it is constructed with the Chebyshev polynomials [19,20]. The *m*th Chebyshev polynomial is given by the following equation [20]:

T_m_ (X) = cos (m acos (x)) (1)

If |x| > 1, the value of acos (x) will be imaginary. Therefore, Equation (1) can be written in terms of hyperbolic cosines: T_m_ (X) = cosh (m acosh (x)).

By setting x = cos θ, or θ = acos (x), T_m_ (x) = cos(mθ). Now, the cos(mθ) can be expanded as a polynomial in powers of cosθ, where the expansion coefficients are the Chebyshev polynomial, shown below:
Cos (0θ) = 1
T_0_ (x) = 1Cos (1θ) = cos θ 
T_1_ (x) = xCos (2θ) = 2 cos^2^ θ − 1→T_2_ (x) = 2x^2^ − 1Cos (3θ) = 4 cos^3^ θ − 3 cos θ
T_3_ (x) = 4x^3^ − 3xCos (4θ) = 8 cos^4^ θ − 8 cos^2^ θ + 1
T_4_ (x) = 8x^4^ − 8x^2^ + 1

The Chebyshev polynomial has equal ripples when |x| < 1, but it increases like x^m^ when |x| > 1.

**Figure 9:**
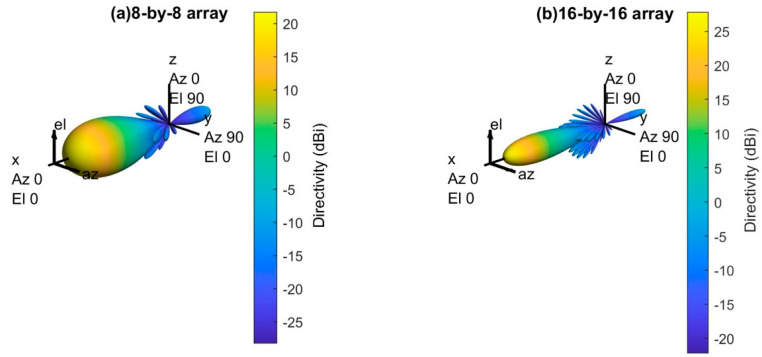
3D radiation pattern of the array with amplitude tapering.

Figure 9 shows the 3D radiation pattern of the uniform phased array antenna (i.e., 8-by-8 and 16-by-16 array) with Dolph–Chebychev amplitude tapering. In this simulation, 30 dB amplitude tapering is applied to reduce the sidelobe power. It is found that the amplitude tapering reduces the sidelobe power significantly for both arrays.

**Figure 10:**
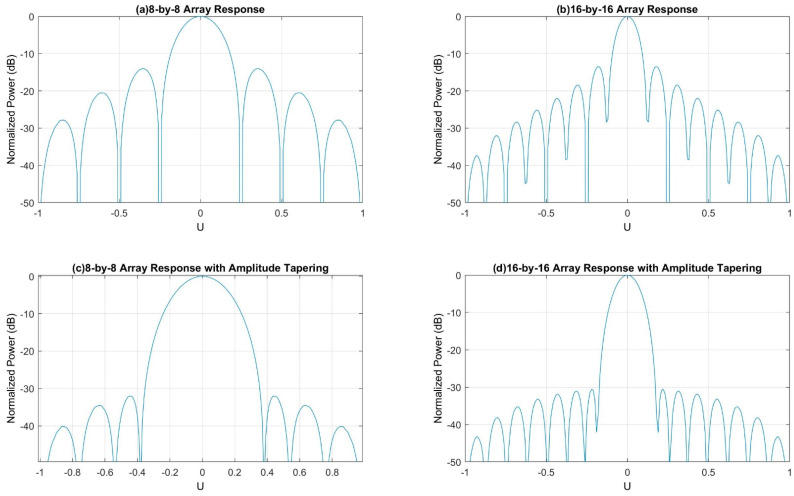
Normalized power distribution of the array (i.e., 8-by-8 and 16-by-16 array) with and without amplitude tapering.

However, the main lobe beamwidth gets wider for both arrays after the amplitude tapering. It will reduce the overall array antenna gain for both arrays. Figure 10 shows the normalized power distribution of the array (i.e., 8-by-8 and 16-by-16 array) with and without the Dolph–Chebychev amplitude tapering. It is found that the sidelobe power reduced significantly for both arrays after amplitude tapering but provided wider beamwidth. 

**Figure 11:**
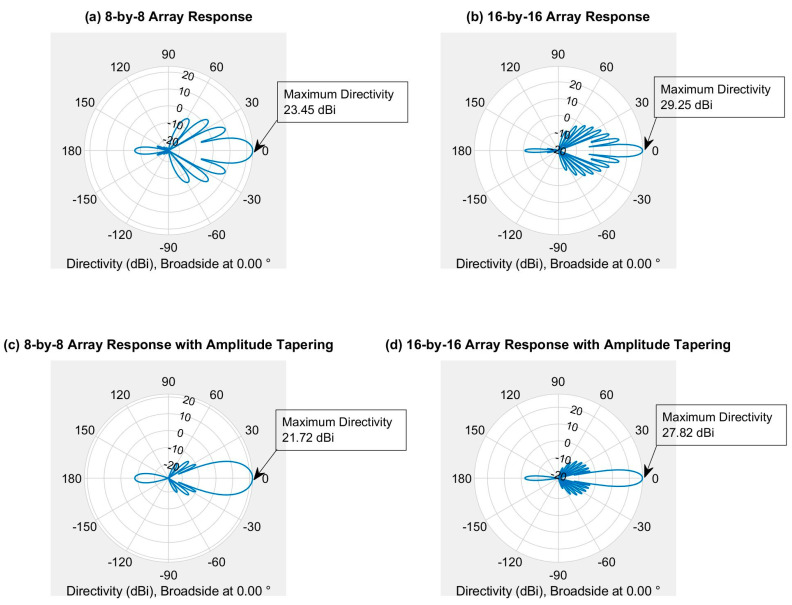
2D radiation pattern of the array (i.e., 8-by-8 and 16-by-16 array) with and without amplitude tapering.

Figure 11 shows the 2D radiation pattern of the array (i.e., 8-by-8 and 16-by-16 array) with and without Dolph–Chebychev amplitude tapering. It is found that the amplitude tapering reduces the sidelobe power significantly for both arrays, but the maximum directivity reduces by 1.73 dBi for 8-by-8 uniform phased array and 1.43 dBi for 16-by-16 uniform phased array. Although both arrays lose small directivity, the maximum power is directed toward the main lobe. Therefore, it will reduce the interferences (i.e., out-of-cell or inter-cell interference) and improves the network performance.

**Figure 12:**
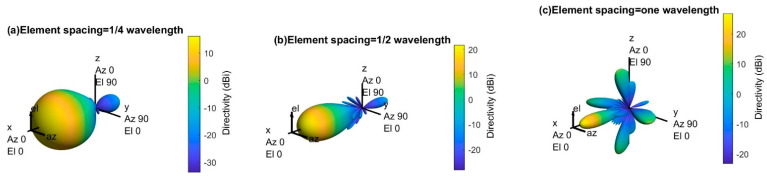
3D radiation pattern of an 8-by-8 antenna array with different element spacing and reduced sidelobe power.

Although the amplitude tapering reduces the sidelobe power, it cannot reduce the grating lobes power, as shown in Figure 12c. Therefore, the element spacing of the uniform phased array must be less than or equal to one-half wavelength to avoid any grating lobes. 

### 2.4. Propagation Modeling

The radiated energy of the transmitter spreads over the surface as it propagates from the transmitter end to the receiver end, and the total signal attenuation depends on lots of factors. Some key factors are listed below [26,27]:

- Operating frequency (e.g., 30 GHz, 70 GHz, etc.);
- Distance between transmitter end and receiver end;
- Cell site location (i.e., indoor or outdoor);
- Propagation environment (e.g., rural, urban, dense urban, etc.);
- Actual terrain (e.g., open, forest, sea, etc.);
- Atmospheric conditions (e.g., rainfall, fog, and clouds, etc.);
- Antenna height (e.g., transmitter height and receiver height).

RF propagation model describes the behavior (in a mathematical form) of the radiated signal that propagates from the transmitter end to the receiver end. It gives an estimation of maximum path loss from the transmitter end to the receiver end, which can be used to estimate the maximum cell range [26,27]. This section describes the basics of propagation modeling for the 5G network. 

#### 2.4.1. Free Space Propagation Modeling

The ideal propagation condition is considered as the free space propagation where the transmitter and receiver will have a direct line of sight without any absorbing or reflecting obstacles. The signal attenuation in the ideal condition called the free space path loss is given by Equation (2) [26].

(2) $$L_{FS}={\left(\frac{\lambda }{4\pi d}\right)}^{2}\;$$

where λ = c/f is the wavelength of the transmitted signal, c is the speed of light (3 × 108 m/s), and f is the operating frequency of the transmitted signal. The propagation path length is represented by d.

**Figure 13:**
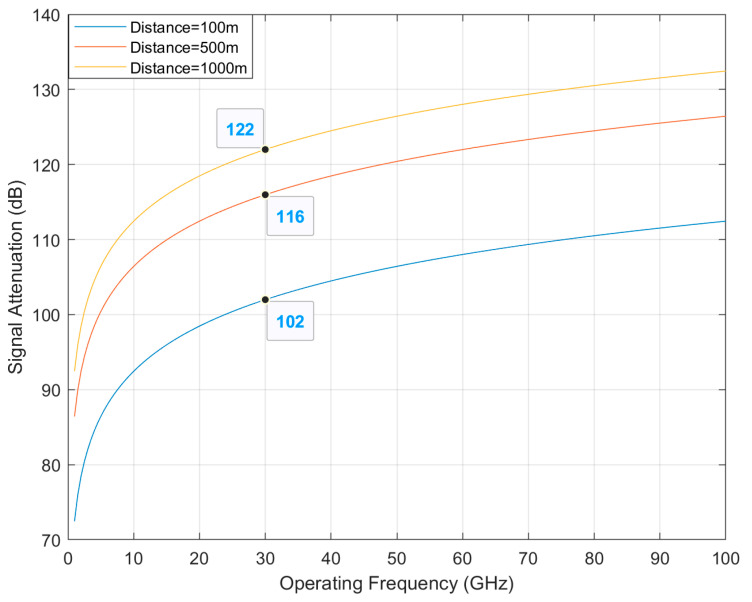
Free space propagation loss for different operating frequency.

Figure 13 shows the total free space signal attenuation versus operating frequency. It is found that the total signal attenuation increases with the operating frequency. The total signal attenuation also increases significantly if the propagation path increases, which is why the 5G networks limit the cell range to 100 m for high-frequency bands.

#### 2.4.2. RF Signal Attenuation Due to Rainfall

Radio Frequency (RF) signal attenuates when it propagates through a region with rainfall. The total RF signal attenuation due to rainfall can be calculated by ITU rainfall model Recommendation ITU-R P.838-3, shown in Equation (3) [28]. This specific attenuation model is valid for frequencies from 1–1000 GHz.

L = d_eff_ k R^α^ (3)

where R is the rain rate in mm/hr, the parameter k and exponent α depend on the frequency, the polarization state, and the elevation angle of the signal path. The effective propagation distance is represented by d_eff_ equal to the geometric distance, *d*, multiplied by a scale factor *r* [28,29].

(4) $$r=\frac{1}{0.477d^{0.633}R_{0.01}^{0.073\alpha }f^{0.123}-10.579\left(1-exp\left(-0.024d\right)\right)}\;$$

where *f* represents the operating frequency.

**Figure 14:**
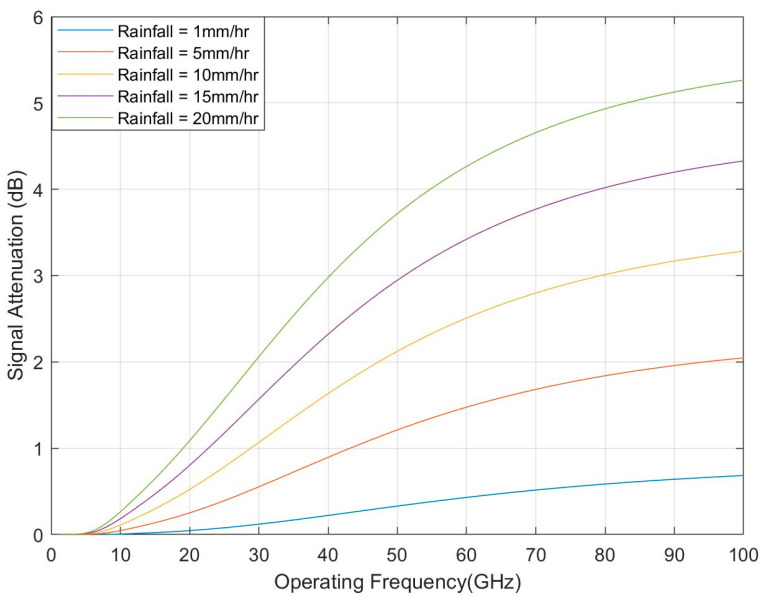
RF signal attenuation due to rainfall for different operating frequency.

Figure 14 shows the total RF signal attenuation versus operating frequency for different rainfall rates. It is found that the signal attenuation increases with increasing the operating frequency. The signal attenuation also increases if the rainfall increases from light rain (i.e., rainfall = 1 mm/hr) to heavy rain (i.e., rainfall = 20 mm/hr). However, the total signal attenuation for the 30 GHz operating frequency will be around 2 dB for the heavy rainfall (e.g., worst-case scenario). This loss can be overcome by increasing the transmit power.

#### 2.4.3. RF Signal Attenuation Due to Fog and Clouds

Fog and clouds are the same atmospheric phenomenon, differing only by height above the ground. RF signal attenuates when it propagates through fog and clouds. The total signal attenuation can be calculated by the ITU model (Recommendation ITU-R P.840-6), shown in Equation (5) [30]. This specific attenuation model is valid for frequencies 10–1000 GHz, and it can be used for both fog and clouds, depending on the height above the ground [30].

L_c_ = K_l_(f) M R (5)

where M is the liquid water density in gm/m^3^, and R represents the propagation path length. The quantity Kl(f) is the specific attenuation coefficient and depends on the operating frequency.

**Figure 15:**
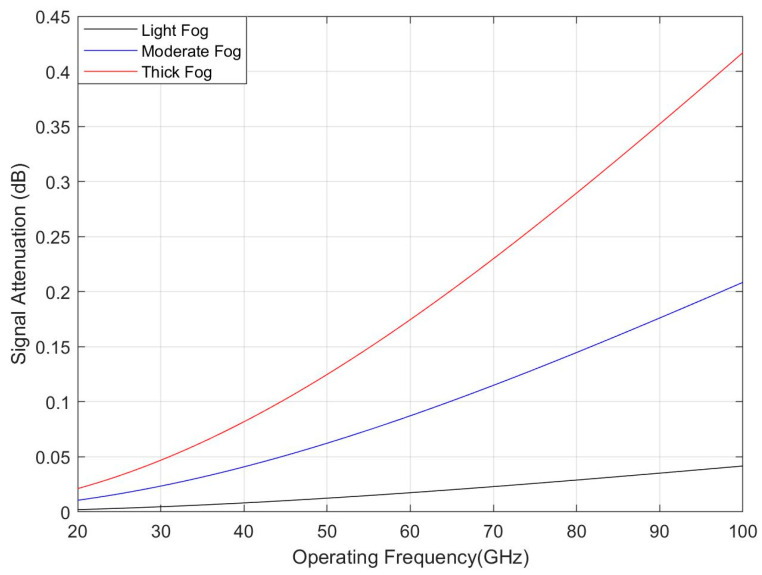
RF signal attenuation due to fog for different operating frequency.

Figure 15 shows the total signal attenuation due to the fog (i.e., light fog to thick fog) for different operating frequencies. It is found that the signal attenuation increases with the operating frequency and the fog densities (i.e., liquid water density is 0.05 g/m^3^ for light fog and 0.5 g/m^3^ for thick fog). However, the total signal attenuation is below 0.45 dB even in the thick fog due to the small propagation path (i.e., cell radius is 200 m).

### 2.5. RF Link Budget Analysis

RF link budget is an equation that accounts for all of the power gains and losses experienced by the RF signal when it propagates from a transmitter to a receiver. It is used to calculate the received signal power required to ensure that the signal arrives at the receiver with adequate signal-to-noise ratio (SNR). A typical RF link budget is shown in Equation (6) [26,27,31]:

P_RX_ = P_TX_ + G_TX_ − L_TX_ − L_FS_ − L_M_ + G_RX_ − L_RX_ (6)

where the P_RX_ is the receive signal power (dBm), and P_TX_ is the transmitted signal power (dBm). The transmitter and receiver antenna gains are represented by G_TX_(dBi) and G_RX_(dBi). L_TX_(dB) and L_RX_(dB) are transmitters and receiver-associated losses (e.g., cable loss, feeder, and connector loss, etc.), respectively. L_FS_ is the free space path loss between the transmitter and receiver. For all other miscellaneous losses (e.g., atmospheric loss, fading margin, body loss, polarization mismatch, etc.) is represented by L_M_(dB). 

Once the antenna parameters (i.e., gain and losses) are known, the transmit power can be adjusted to overcome the losses and meet the required SNR [26]. This paper uses the 5G network specifications of IMT-2020 for 30 GHz operating frequency [16].

RF link budget computation is not complete until the required SNR (i.e., ε_b_/N_0_) is specified to keep the error rate under a given threshold and maximize the network performance (i.e., maximum data rate). The required SNR can be calculated by Equation (7) [26]:

(7) $$\frac{\varepsilon_{b}}{N_{0}}=\frac{1}{R}\frac{P_{RX}}{N_{0}}$$

where (ε_b_/N_0_) is the required signal-to-noise ratio (SNR) per bit, R is the propagation path length, P_RX_ is the receive signal power, and N_0_ is the thermal noise at the receiver. 

Once the required SNR is known from the 5G network specification (i.e., IMT 2020 [16]), the required receive signal threshold can be calculated for a certain propagation path length by using Equation (7). 

## 3. 5G Network Coverage and SINR Simulations

The free-space propagation loss is high in the millimeter-wave spectrum (e.g., 30 GHz), as shown in Section 2.4. Due to a very small wavelength, it also suffers a high probability of signal blockage (e.g., the leaves on trees can block the signal). Therefore, the 5G network in high-frequency bands (i.e., millimeter-wave spectrum) will require a direct line of sight connection between the cell site and user (e.g., mobile phone) for the optimal network performance [32,33,34]. Hence, this paper uses the free space propagation model to estimate the 5G network coverage. However, the actual 5G network coverage can dramatically vary with the deployment location, as explained in Section 2.4. 

**Figure 16:**
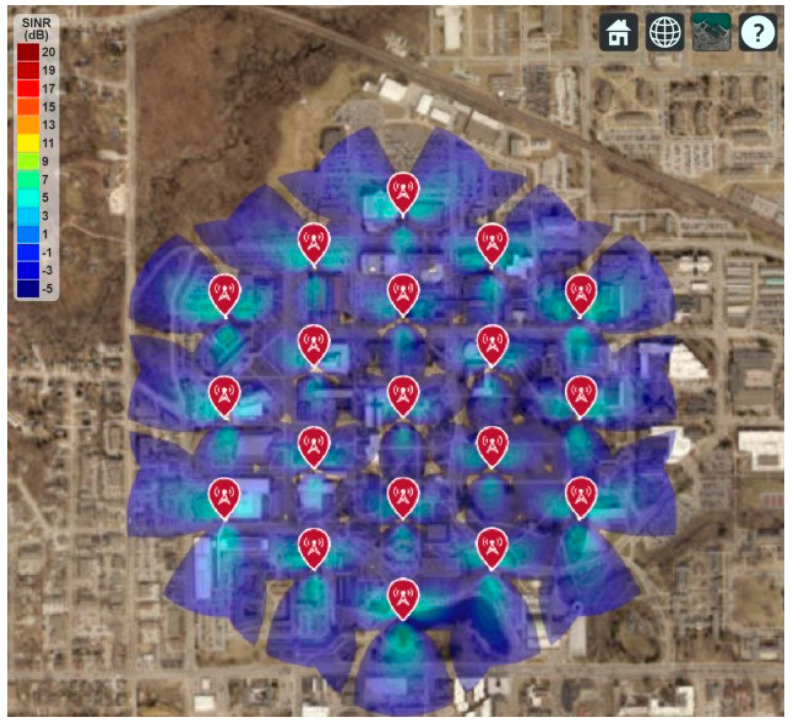
5G Network Coverage and SINR Map for a single antenna element.

**Figure 17:**
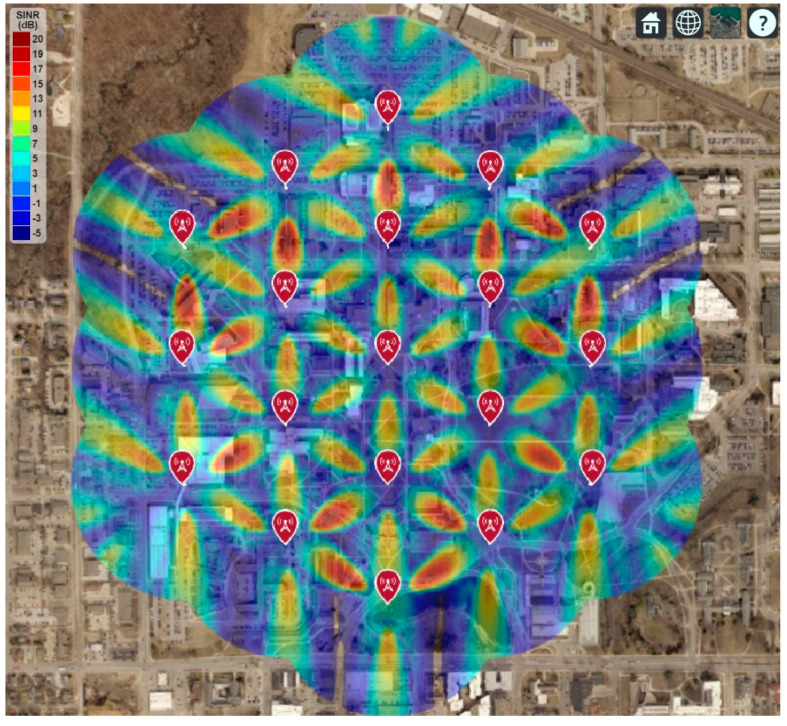
5G Network Coverage and SINR Map for 8-by-8 Antenna Array.

This section shows the real-world 5G network coverage simulation and RF signal tracing where the Iowa State University is considered as the deployment area. The Google map is used to display the simulated network coverage and the signal-to-interference-plus-noise ratio (SINR), shown in Figure 15, Figure 16 and Figure 17. This paper follows the guidelines of IMT-2020 for the 5G network planning and coverage simulation [16]. All the simulation parameters are listed in Table 2. 

Figure 16 shows the 5G network coverage and SINR for a group of 19 cell sites, each with three sectors, and each sector has a single antenna element. It is found that the maximum SINR in the deployment area is below 11 dB. It is also found that some areas do not have the minimum required SINR, which will create a coverage gap for the user. Therefore, single antenna elements for each transmitter in 30 GHz operating frequency will not be an ideal option.

However, the RF coverage improved when each transmitter used an 8-by-8 array instead of single antenna elements, as shown in Figure 17. It is found that the maximum SINR improves to 17 dB. Although the average SINR is still not good throughout the deployment area, most areas now have the RF coverage for the 5G network. 

**Figure 18:**
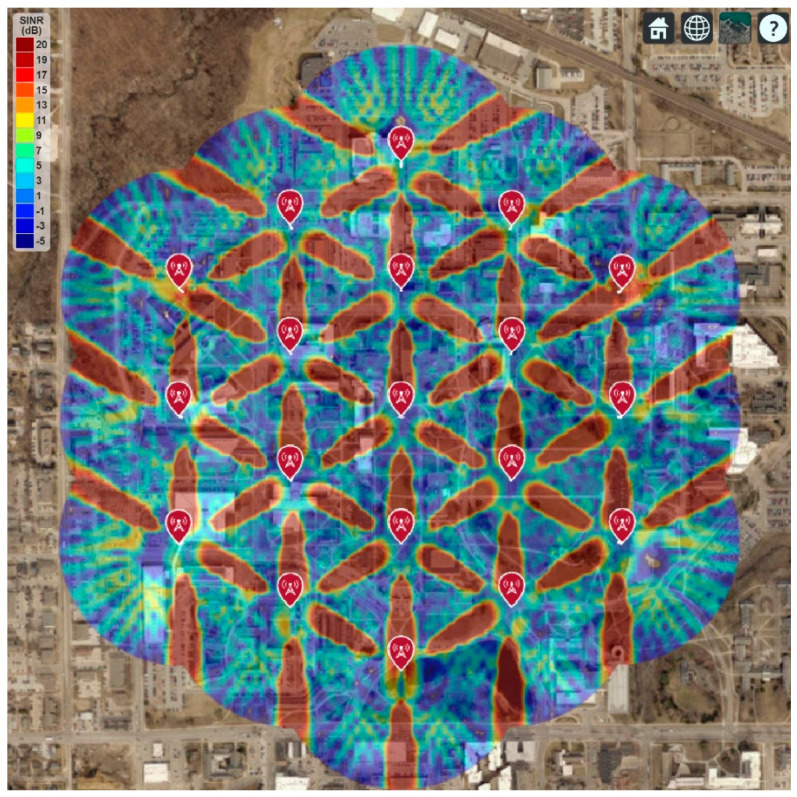
5G Network Coverage and SINR Map for 16-by-16 Antenna Array.

The best RF coverage is achieved by adding a 16-by-16 array antenna for each transmitter, as shown in Figure 18. Most of the deployment areas now have 19 dB or higher SINR, which is required for the 5G network. Since the array gain (i.e., directivity) increases as the number of elements increases in the array, the peak SINR also improves as expected.

For optimal performance (e.g., 99.99% network availability, 99% network reliability, maximum data rate, etc.), the 5G network will require high SINR, which can be achieved using the larger size phased array antenna for each cell site. The phased array antenna also allows changing the phase of the individual elements of the array. It can be used to steer the transmitted signal in any particular direction to improve the 5G network coverage. 

However, the 16-by-16 array provides a highly directional and narrow radiation beam, shown in Section 2. It causes maximum SINR toward the direction of each sector but minimum in all other directions, as shown in Figure 18. Thus, the average SINR will significantly vary throughout the deployment area, impacting the overall system performance. Therefore, Section 4 proposes an updated cell site architecture to improve RF coverage, including higher-order sectorization and adjusting antenna parameters (e.g., antenna downtilt, antenna height, etc.).

## 4. 5G Network Coverage and Signal Strength Simulations

This section provides RF coverage and signals strength simulation for 30 GHz operating frequency with different transmitter parameters (e.g., array size and downtilt angle). All the simulation parameters are listed in Table 2, except the number of cells is 7 instead of 19. 

### 4.1. RF Coverage and Signal Strength

**Figure 19:**
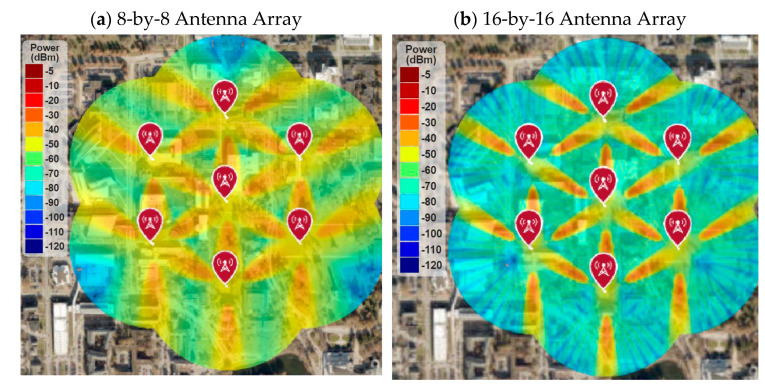
RF coverage and signal strength for 30GHz operating frequency.

Figure 19 shows the RF coverage and signal strength simulations of seven cell sites, each with three sectors. It shows the RF coverage comparison of an 8-by-8 array with a 16-by-16 array. It is found that the 8-by-8 array provides excellent signal strength (i.e., higher than −50 dBm) throughout the deployment area, but the 16-by-16 array provides very poor average signal strength throughout the area. Some areas that are in the line of sight of each sector get the maximum RF signal (i.e., higher than −40 dB) due to the highly directional antenna, but most of the areas will not have the minimum received signal level to meet the network requirements. 

Although the 5G network will support up to 16-by-16 array for 30 GHz operating frequency, the traditional three-sector cell architecture will not provide better RF coverage and SINR due to the highly directional antenna beam. Therefore, this paper proposes higher-order sectorization for advanced arrays, where each cell will have six sectors instead of three. The simulation results are shown in Section 4.2. 

### 4.2. Improve RF Coverage with Higher-Order Sectorization

**Figure 20:**
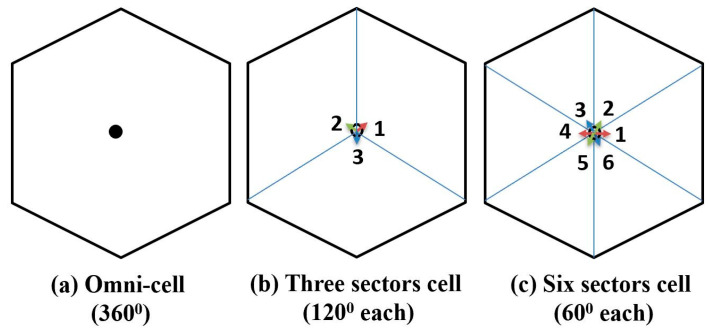
Cell sectorization with directional antennas.

The transmitted beamwidth is very wide in the lower frequency spectrum (e.g., 4G network), with a smaller array antenna (e.g., 4-by-4 array) for each transmitter site. It is useful to cover a wide area, but it also limits the number of sectors per cell site due to the poor carrier-to-interference (C/I). The higher-order sectorization (e.g., six-sector and nine-sector cell) is only possible if we use narrow beam and highly directional antennas that offers high C/I. Since the 16-by-16 array provides a very narrow and highly directional beam, we can use six sectors per cell instead of three. Figure 20 shows the cell architecture with omni-directional antennas, three sectors (120° each), and six sectors (60° each). 

**Figure 21:**
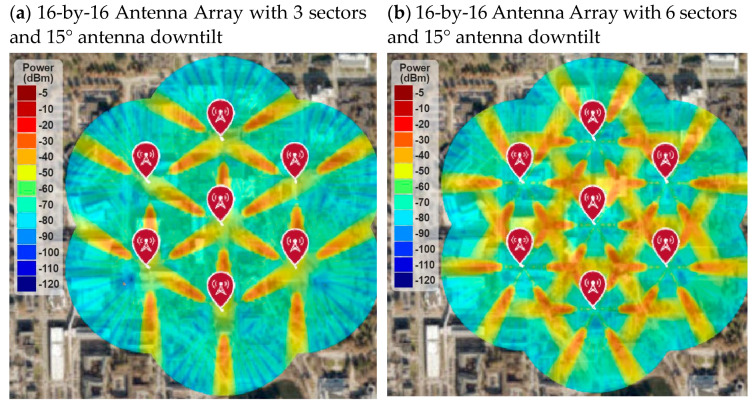
RF coverage and signal strength versus higher-order sectorization.

Figure 21 shows the RF coverage and signal strength simulation results of a 16-by-16 array with three-sector and six-sector cells. It is found that the average signal strength improves by adding more sectors to each cell, as shown in Figure 21b. The average signal strength is higher than −50 dBm in most of the area. However, the signal strength is still not uniformly distributed, and some areas still do not have a suitable RF signal level. To optimize the RF coverage, Section 4.3 shows RF coverage simulation with different antenna downtilt angles.

### 4.3. Improve RF Coverage with Appropriate Antenna Downtilt Angle

**Figure 22:**
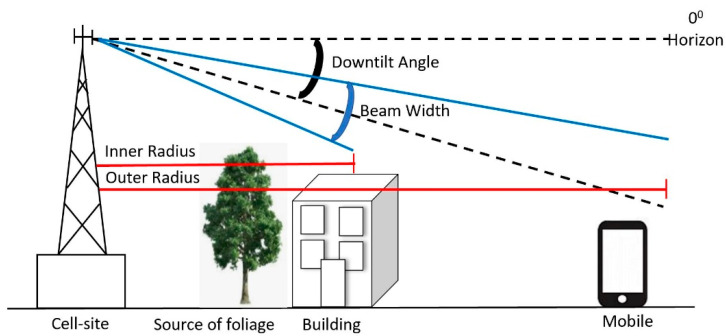
RF coverage with antenna downtilt.

**Figure 23:**
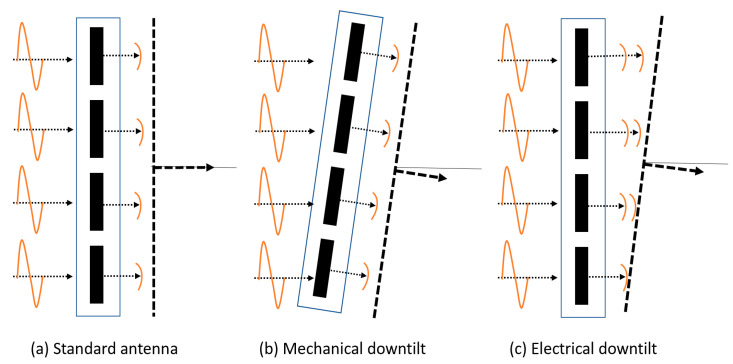
Types of antenna downtilt.

The BS antenna downtilt is one of the most effective ways to control the radiated beam [35]. A typical downtilt scenario is shown in Figure 22. The effect of downtilt in RF coverage and signal strength is discussed in this section. The system designer can use mechanical (e.g., physically adjusting each antenna element pole mount) or electrical downtilt (e.g., changing the phase of each element electrically) to tilt the radiated beam, as shown in Figure 23. This paper uses electrical downtilt.

Since this paper uses a uniform phased array antenna for each transmitter, the radiation beam can be tilted to any desired direction by adjusting the phase of each antenna element. The simulation results shown earlier in this section use antenna downtilt angle 15°, but this section shows the effect of different downtilt angles on RF coverage and signal strength. 

**Figure 24:**
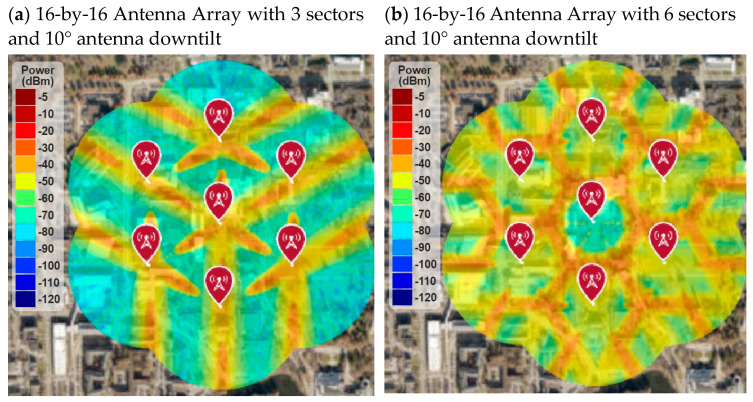
RF coverage and signal strength versus antenna downtilt angle.

Figure 24 shows the RF coverage and signal strength simulation for 16-by-16 antenna array with multi-sectors cell and 10° antenna downtilt instead 15° antenna downtilt. It is found that the RF coverage improved for both three-sector and six-sector cells. However, the six-sector cell architecture with 10° antenna downtilt provided the best RF coverage and signal strength. The average RF signal strength was higher than −50 dB throughout the deployment area, which is a very good RF signal for the 5G network. 

**Figure 25:**
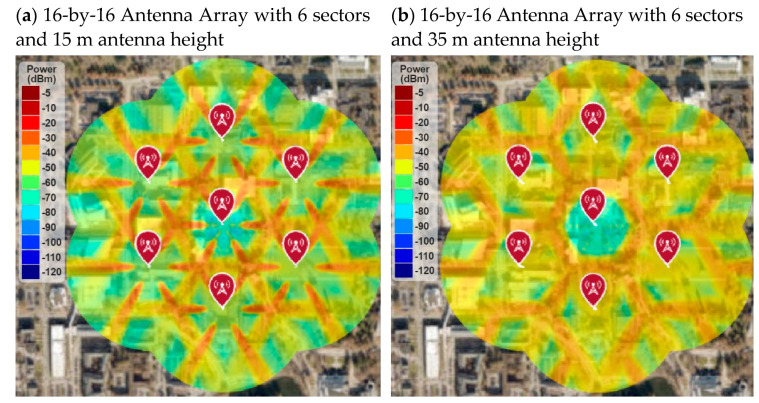
RF coverage and signal strength versus antenna height.

In addition, the BS antenna height also impacts the RF coverage. Figure 25 shows the simulation results with changing the antenna height and keeping all the simulation parameters the same as Figure 24b. Results show that the RF coverage degrades significantly if antenna height reduces from 25 m to 15 m, as shown in Figure 25a. The RF coverage is better in 35 m antenna height than 15 m, but it is still not as good as 25 m.

In summary, the traditional cell architecture needs to evolve from three-sector cells to six sectors or higher sectors in a higher frequency spectrum (e.g., 30 GHz) to provide better RF coverage and signal strength. The common antenna parameters will also play a key role in the RF coverage planning, including antenna downtilt angle, antenna height, and transmit power. Therefore, the system designer needs extensive studies and testing to find the optimum number of sectors per cell, antenna downtilt angle, antenna height, and required transmit power.

This paper uses the terrain profile from Google Maps. The RF signal tracing shows in the simulation results dependent on the updated Google maps. As long as Google Maps updated the terrain profile, the simulation results can be comparable to the real-world deployment. However, the system designer can obtain the updated city map (i.e., terrain profile) from the city’s local government. 

## 5. 5G Network Deployment Challenges

Unlike earlier generation networks (e.g., 4G and 3G), uniform cell coverage planning will not be an option for 5G network deployment. The 5G network coverage planning will heavily depend on the individual cell site location (e.g., urban, suburban, and rural). This section describes potential challenges for 5G network coverage planning and deployments. 

### 5.1. Accurate Site Planning

The 5G network will not be a standalone deployment at the initial stages of the network development. It will utilize all the existing cell sites (i.e., 3G/4G sites) as the candidate sites for 5G networks [36]. Since the propagation loss is high in the millimeter wave spectrum, it will limit the 5G cell radius to 100 m compared to several kilometers in the 4G network, as discussed in Section 3. To meet the high availability (i.e., 99.99% availability) requirements of 5G networks, MNOs will need to deploy hundreds of small cells compared to one large 4G cell. The following are the key limiting factors that reduce the viable cell site locations that impact accurate cell site planning:

1. Suitability: The main challenges are associated with the availability of space on the premises. It can fit all the necessary transmitter equipment and underground space to add a new chamber for the cell site. The availability of deliverable power and total ownership cost will also decide factors to choose a cell site location. Finally, the propagation characteristics and the availability of neighboring cell sites to maintain the uniform RF coverage through the deployment area.
2. Accessibility: It involves the challenges of acquiring the cell site locations. It includes a lengthy process of approval from the private owner to the local government.

However, all the cell sites must be considered together while RF coverage planning to reduce redundant sites, out-of-cell interference, and deployment costs. Hence, accurately planning hundreds of cell sites in a particular deployment area and meet all the 5G network requirements will be a big challenge for the MNOs.

### 5.2. Acquiring Cell Site Location

The 5G network does not require a tall and large structure to deploy 5G cell site, unlike the current generation network (e.g., 4G). It can be deployed in an existing structure (e.g., edges of the buildings or the lamp post beside the road, etc.) in any deployment area. Identifying the appropriate cell site location will be the key for a uniform RF coverage that meets all the 5G network requirements. Since the MNOs will need to deploy a massive number of new cell sites for 5G network, they will need to acquire hundreds of new small cell locations compared to one large cell location for 4G network. Acquiring these massive number of cell site locations will significantly increase the 5G network deployment cost and the operational expenditure (OPEX) [37,38]. In addition, there are some key restrictions and challenges of acquiring a massive number of new cell sites, listed below:

1. Government approval policy: The MNOs need approval from the local government for every cell site installation and sign a lease agreement. According to some operators, the new small cell deployment requires 18 to 24 months and maximum delay caused by the government approval process [39]. Since safety and local heritage always remain the local government’s key consideration, it requires high fees and extensive maintenance of the public property.
2. Private owner agreement: When a new cell site needs to deploy in a privately owned property (i.e., residential or non-residential), the MNOs will need more than just approval from the regulators. Since the rent valuation is not fixed countrywide, it might take months to achieve rental fee agreement between both parties. It will increase the cell deployment time. The private owner must be compensated, but the excessive rent-seeking can add unnecessary delay in the cell deployment process.
3. Multi-operator case: There might be several operators countrywide or worldwide (e.g., Verizon and AT&T in the USA) who want to provide 5G services. Since every operator will want to develop their own 5G network to dominate the market, the number of new cell sites will grow exponentially in any particular deployment area. Therefore, it will become a new issue for the local government to approve a new cell site in the current structure or build a new structure in any public or privately owned area [40,41].

Since different parties (e.g., MNOs, private owners, local and national government, etc.) are involved throughout the deployment process, appropriate regulations and frameworks will be required. Some guidelines are listed below:

1. The government needs to play a key role in keeping the balance of all parties’ interests. The regulator should include the rules and timeline that must be followed by all parties to avoid unnecessary delay.
2. The government must provide clear guidelines about fees payable for a new cell site or renewal of an old cell site.
3. All the rules and regulations established by the local government and the local authorities must be precise and shared with the operator in precise language.
4. The local government should have clear guidelines for private owners about rental valuation to avoid excessive rental value.
5. A clear regulation is required for multi-operator and shared resources to avoid the high cost to the operator.

### 5.3. Propagation Modeling and Coverage Prediction

The millimeter-wave spectrum has higher propagation loss, discussed in Section 3. Since the RF signal characteristics greatly vary with the deployment area, the MNOs will need to analyze the signal characteristics through a medium and estimate the signal parameters. However, the actual cell site measurement requires significant time and additional resources that increase the operational cost for the MNOs. For example, drive testing and RF signal measurement throughout the deployment area require a lot of manpower, equipment, and operational costs. On the other hand, propagation modeling provides a low-cost, suitable, and convenient alternative to estimate the RF signal characteristics in any deployment area. It provides estimated RF coverage and radio signal characteristics before deploying and testing the RF signal through drive testing around a cell site. The system designer can use this information to identify the accurate cell site location with appropriate cell site parameter including required transmitted power, antenna height, BS antenna downtilt angle, power supply, and backhaul connection, etc.

However, the current empirical propagation modeling does not apply to the millimeter-wave spectrum. For example, the 4G network uses the Okumura–Hata propagation model, and it applies from 150 MHz to 2000 MHz based on the different deployment scenarios (e.g., rural, suburban, urban, and dense urban) [27,31]. Thus, it will impose a new challenge, and the system designer needs to develop a new propagation model for the millimeter-wave spectrum. 

Several research works have already been published on 5G propagation modeling [42,43,44]. This paper shows the real-world 5G network coverage analysis using MATLAB and Google Map tools with free space propagation modeling. Since the terrain profile is used from the Google map, the accuracy of the simulation results depends on the updated Google maps. However, the system designer can obtain the local government’s updated city map for accurate RF coverage and signal analysis. 

### 5.4. Challenges Raised by 5G New Radio

Massive MIMO (i.e., multiple-input and multiple-output) will be one of the key enabling technologies for the 5G networks to increase the network capacity, increase the data rates, enhance the network reliability, improve the energy efficiency, and reduce the interferences [36,45,46]. However, massive MIMO will significantly change traditional network planning based on multiple sectors per site with wide antenna beams. Massive MIMO uses antenna arrays (e.g., 16-by-16 array shown earlier) for simultaneous transmission and reception. As we increase the array size, the antenna beam is no longer a fixed wide beam; rather, it will be narrower and highly directional. The traditional network planning will not meet the massive MIMO requirements on planning, coverage prediction, data rate, and capacity [36]. Further study is needed to identify and overcome all the implementation challenges for the efficient implementation of massive MIMO.

### 5.5. Energy Efficiency

**Figure 26:**
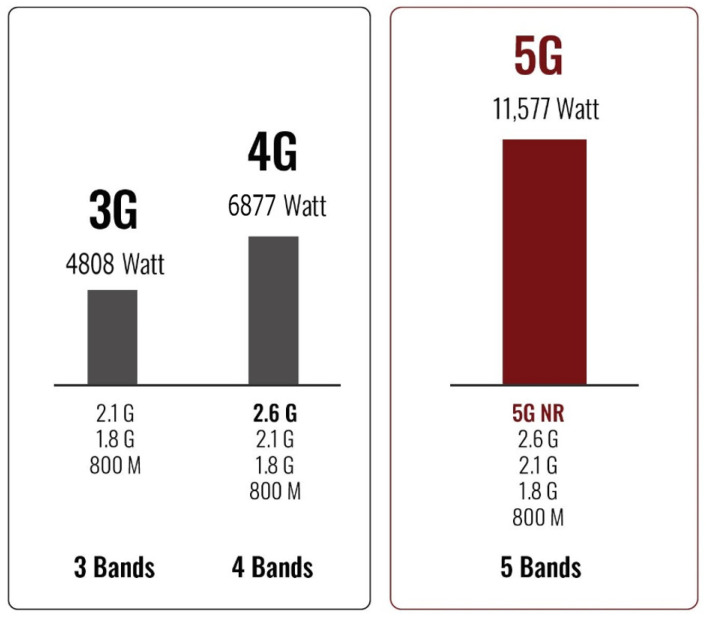
Cell site power consumption.

Figure 26 compares maximum power consumption/site for 3G, 4G, and 5G, respectively. It is shown that the 5G cell site will require 11.5 kW of power compared to 6.8 kW for the 4G network [47]. It is also estimated that 4.4 terawatt-hours (TWh) of power were consumed by about 100 million small cells in 2020, and the total power consumption in the ICT sector may reach up to 1700 TWh by 2030 [48,49]. This motivates the researchers to focus on green communications to develop energy-efficient solutions for 5G networks with a target of reducing energy consumption up to 90% [50,51].

Since a massive number of new cells will be deployed for the 5G network, the total power requirements will increase exponentially [52]. This will bring new challenges for the MNOs that need to be addressed:

1. To deploy a massive number of new cell sites will require additional power in the deployment area, but the total power supply is limited for a given area (i.e., any area in a city). It will limit the availability of power for individual cell sites, which will impact the overall network planning.
2. Since the power grids are limited in power supply, they require significant time (~up to a year) to increase the capacity. Therefore, the MNOs have to deal with this additional delay for the cell site development.
3. Since the electricity cost is high, it will increase the overall operational cost of the network.

Some guidelines to overcome these challenges are listed below:

1. All the network-related equipment must be optimized in terms of energy efficiency.
2. Develop an advance control unit to reduce the static power consumption when there is no traffic.
3. Find an alternate power source (e.g., solar cell, battery) to meet extra power requirements.

### 5.6. Backhaul Network

The advancement of consumer electronics and the availability of the network connection drives the ever-growing traffic worldwide. According to the ABI Research report, the estimated data traffic will exceed 2708 exabytes in 2025. Therefore, the 5G network will use a higher frequency spectrum (e.g., millimeter-wave) that offers higher bandwidth (i.e., ~1 GHz). However, the cell radius is limited to 100 m due to high propagation loss in the millimeter wave spectrum. Therefore, the MNOs need to deploy a massive number of new cells compared to one large cell for 4G. It will boost the network capacity and data rate several fold [53]. 

However, each cell site will need direct connection with the core network for sharing the radio resources, called the backhaul network [54]. It will bring new challenges for the MNOs that need to address:

1. The cost factor is high to deploy a new backhaul connection. The wired backhaul (i.e., fiber connection) will be the priority for the MNOs due to the high capacity, but it is not a cost-efficient solution and requires a significant amount of deployment time. The wireless backhauls solutions (e.g., microwave backhaul) are cost-efficient and require less deployment, but the current microwave backhaul is limited by capacity, and it will not be able to support high capacity. Therefore, the MNOs need to find a cost-effective backhaul solution to support the massive number of new cells.
2. One-hop direction connection with the core network provides a low latency communication link. However, it is no longer feasible for the 5G network due to small antenna height (i.e., 25 m), unlike the 4G network. Since a 5G network requires very low latency, it will be a big challenge for the MNOs to deploy a massive number of new cells and meet this requirement [55,56].
3. Since each cell site requires additional equipment for the backhaul solution, it will increase the power requirements/cell site.

## 6. Conclusions

The 5G network in the higher-frequency spectrum will require 100% network coverage to meet all the key performance indicators (KPI) (e.g., 99.99% availability, 99.99% reliability, highest data rates, etc.). However, the network performance can degrade significantly for inaccurate coverage estimations: (1) underestimating coverage that can cause overlapping coverage with the neighboring cells, which will increase the out-of-cell interferences and (2) overestimating coverage that can cause the received signal level below the required threshold at the cell edge. Therefore, accurate RF coverage planning and RF signal analysis will be the key priorities of the MNOs to ensure 100% network coverage.

This paper describes the 5G network coverage planning, including the transmitter site design with uniform phased array antenna, network layout design with a group of cells, propagation modeling, RF link budget analysis, and implementation of higher-order sectorization with appropriate antenna downtilt angle. It is found that the higher-order sectorization with advance phased array antenna provides better RF coverage in a higher frequency spectrum. This paper also describes the real-world deployment challenges (e.g., accurate cell site planning, acquiring thousands of new cell site locations, energy efficiency, backhaul, etc.) that must be overcome for the faster 5G network deployment.

## Figures and Tables

**Table 1:** Evolution of technology generations in terms of services.

Generation	Year	Primary Services	Key Differentiator
5G	2020s	Massive mobile broadband, massive Internet of Things (IoT), industrial automation	Digitalization of industry, ultra-low latency, ultra-high availability, ultra-speed, and ultra-reliability
4G	2010s	All 3G services and mobile video consumption with higher data speed	Faster broadband internet and lower latency
3G	2000s	Phone calls, messaging, mobile web browsing	Broadband internet and smartphones
2G	1990s	Mobile voice calls and messaging	Digital
1G	1980s	Analogue voice calls	Mobility

**Table 2:** Simulation parameters for 5G network coverage and SINR.

Parameters	Value
Operating Frequency	30 GHz
Bandwidth	80 MHz
BS Antenna height	25 m
BS Transmit power	40 dBm
BS Antenna gain	10 dBi
BS Antenna noise figure	7 dB
Receiver height	1.5 m
Receiver noise figure	7 dB
Receiver gain	8 dBi
No. of cell sites	19
Inter-site distance	200 m

## Data Availability

No new data were created or analyzed in this study. Data sharing is not applicable to this article.
